# Supplementary material for: MetaRibo-Seq measures translation in microbiomes
Source: Nat Commun. 2020 Jun 29;11:3268. doi: 10.1038/s41467-020-17081-z (PMC7324362; doi:10.1038/s41467-020-17081-z)
Supplement: Supplementary file 10 — Supplementary Data 7 [file 41467_2020_17081_MOESM10_ESM.zip › File2/Confidence_High_Taxonomy/135713_out.krona.html]

Javascript must be enabled to view this page.

members
magnitude
magnitudeUnassigned
count
unassigned
taxon
rank

135713\_out

3

3
2759
superkingdom

3
kingdom
4751

451864
subkingdom
3

1
3
phylum
4890

SRS019685\_contig\_number\_12237

subphylum
147537
2

class
4891
2

order
4892
2

2
4893
family

2
33170
genus

2
species

SRS015190\_contig\_number\_contig-100\_2039.96342SRS043411\_contig\_number\_1445
33173
